# Heterologous Overexpression and Functional Analysis of the *Isodon suzhouensis IsKS1* Gene in *Arabidopsis thaliana*

**DOI:** 10.3390/cimb47060413

**Published:** 2025-06-03

**Authors:** Fawang Liu, Kefeng Zhai, Dongmei Xie

**Affiliations:** 1 School of Biological and Food Engineering, Suzhou University, Suzhou 234000, China; fawang10314@ahszu.edu.cn; 2 Engineering Research Center for Development and High Value Utilization of Genuine Medicinal Materials in North Anhui Province, Suzhou 234000, China; 3 Anhui Provincial Engineering Laboratory for Efficient Utilization of Featured Resource Plants, Huaibei 235000, China; 4 Anhui Province Key Laboratory of Research & Development of Chinese Medicine, Hefei 230012, China; xiedongmei@ahtcm.edu.cn

**Keywords:** *Isodon suzhouensis*, kaurene synthase, transcriptomic and metabolomic, wangzaozins

## Abstract

*Isodon suzhouensis,* also known as “Wangzaozi”, is an edible and medicinal plant belonging to the Lamiaceae family. Its main functional constituents are the tetracyclic diterpenoids known as wangzaozins. Wangzaozins have a strong structural similarity to gibberellins (GAs), which are synthesized via the diterpenoid biosynthetic pathway (map00904). The formation of the diterpenoid skeleton is regulated by copalyl diphosphate synthase (CPS) and kaurene synthase (KS). In order to identify and study the *KS* gene involved in wangzaozins biosynthesis, a transcriptomic and metabolomic analysis of *Isodon suzhouensis* was performed. The *IsKS1* gene, which was highly expressed in leaves, was successfully cloned. The binding mode and sites of IsKS1 with its catalyzed substrate, ent-copalyl diphosphate (ent-CPP), were predicted using AutoDock. The docking results revealed hydrophobic interactions, hydrogen bonds, and salt bridges between them. Furthermore, overexpression of *IsKS1* in *Arabidopsis thaliana* resulted in a significant increase in gibberellin content, as well as the up-regulation of *GA2(KS)* and *GA3OX1* genes. These results suggest that the *IsKS1* gene is involved in gibberellin biosynthesis and may potentially contribute to the biosynthesis of wangzaozins.

## 1. Introduction

*Isodon suzhouensis*, is a national geographical indication plant in China that belongs to the Lamiaceae family. It was first mentioned in the Ming Dynasty book “Edible Herbs and Other Plants Needed in Disaster Relief” and was referred to as “Xiangchacai” [1]. *Isodon suzhouensis* has a variety of medicinal properties, including heat-clearing and detoxifying, reducing dampness and swelling, promoting blood circulation, and dispersing blood stasis. It has traditionally been used to treat diseases such as damp-heat jaundice, sore throat, lung abscess, ulcers, tumors, venomous snake bites, and tuberculosis. Modern pharmacological studies have also shown that *Isodon suzhouensis* has pharmacological effects such as anti-tumor, anti-inflammatory, antibacterial, antioxidant, hepatoprotective, and hypoglycemic properties [2]. The main active constituents of *Isodon suzhouensis* are diterpenes, triterpenoids, and flavonoids. The diterpenes, particularly the wangzaozins (wangzaozin A, glaucocalyxin A, and glaucocalyxin B), have significant anticancer activity [3,4].

Wangzaozins have a similar structure to the plant hormone gibberellin (GA), as both are tetracyclic diterpenoids. The biosynthetic pathway of GA has been extensively studied in plants, and the genes responsible for GA biosyntheses, such as *CPS* (copalyl diphosphate synthase) and *KS* (kaurene synthase), have been successfully cloned in *Arabidopsis thaliana* and other plant species [5]. It has been reported that plants treated with excessive GA hormones exhibit severe phenotypes of GA excess, such as increased hypocotyl length, flattened leaves, slender petioles, increased stem height, and earlier flowering time [6]. In *Arabidopsis thaliana*, *AtKS* has a highly tissue-specific expression pattern and serves as a potential regulatory point for controlling GA biosynthesis. Further research has shown that the co-expression of *AtCPS* and *AtKS* in *Arabidopsis thaliana* may promote the production of ent-kaurene, a precursor of tetracyclic triterpenoids, resulting in an increase in bioactive GAs, and ultimately lead to GA excess morphology [7].

*Isodon rubescens* (Hemsl.) H. Hara and *Isodon suzhouensis* belong to the Lamiaceae family and have very similar leaf and stem morphology. In 2022, the genome of *Isodon rubescens* was sequenced, revealing a genome size of 406.8 Mb, which is similar to the 557.2 Mb of *S. miltiorrhiza*. Additionally, key genes of the CYP706 family, such as CYP706V oxidases involved in the skeletal modification of kaurene, were identified, along with an expansion of *CPS* (*IrubCPS1*, *IrubCPS5*) and *KS* (*IrubKS4*, *IrubKS7*, *IrubKS8*, *IrubKS9*) related to the biosynthesis of normal CPP-derived diterpenoids [8]. In a previous study, we investigated the function of wangzaozins in ameliorating COPD progression via the SOCS3-JAKs/STATs pathway [9]. To further understand the genes involved in wangzaozin biosynthesis, we assessed the genome size of *Isodon suzhouensis* using a flow cytometer, which resulted in a genome size of 430 Mb, similar to *I. rubescens*. We also compared the *CPS* gene of *Isodon suzhouensis* with *IrubCPS*, showing a 98% similarity in gene sequence, indicating a high evolutionary relationship between them. Although the activity of IrubKS has been detected in *Isodon rubescens*, little is known about the *KS* gene in *Isodon suzhouensis*, and the activity and function of *KS* in *Isodon suzhouensis* have not been reported.

Based on the common biosynthetic pathway shared by the diterpenoid wangzaozins and GA in *Isodon suzhouensis*, this study utilized transcriptomics and metabolomics to analyze the tissue differences of wangzaozins and investigate genes related to the content variations. Additionally, a *KS* gene involved in the synthesis of the diterpenoid skeleton was identified, cloned, and bioinformatically analyzed. The *IsKS1* gene was overexpressed in *Arabidopsis thaliana*, and its effects on morphology, ent-kaurene, and GAs content were examined to gain preliminary insights into its function. Overall, this research provides valuable information on the function of the *KS* gene in *Isodon suzhouensis* through gene cloning and transgenic studies.

## 2. Materials and Methods

### 2.1. Plant Materials

Young *Isodon suzhouensis* plants were collected from Jiagou Town in Suzhou City, China, and then transplanted into the laboratory of the Engineering Research Center for Development and High Value Utilization of Genuine Medicinal Materials in North Anhui Province. The plants were grown at a temperature of 23 °C, with 80% relative humidity and a light intensity of 350 µmol m^−2^s^−1^. Fresh leaf and stem tissues from both *Isodon suzhouensis* and *Arabidopsis thaliana* were quickly frozen in liquid nitrogen for RNA isolation, sequencing on an Illumina NovaSeq 6000 sequencer, and metabolite detection using Waters ACQUITY UPLC I-Class plus instruments.

### 2.2. Analysis of the Metabolome of Isodon Suzhouensis

The stems and leaves of *Isodon suzhouensis* were crushed and extracted with 500 µL of 70% methanol, containing internal standards of L-2-chloroalanine and succinic acid-d4 at a final concentration of 4 μg/mL. The samples were then ultrasonicated for 30 min, and the extraction was used for LC-MS/MS analysis. HPLC analysis was performed on a Waters ACQUITY UPLC I-Class Plus equipped with an ACQUITY UPLC HSS T3 column (100 mm × 2.1 mm, 1.8 μm). The injection volume was 3 μL, and the mobile phases were solvent A (0.1% formic acid in water) and solvent B (acetate) with an elution gradient of 0–15 min (B, 5–100%). The MS conditions included a spray voltage of 3800 V, a capillary temperature of 320 °C, an aux gas heater temperature of 350 °C, an aux gas flow rate of 8arb, an MS/MS resolution of 17,500, and a mass range (*m*/*z*) of 70–1050. Metabolites were identified by comparing MS data to compounds in the Human Metabolome Database (HMDB), Lipidmaps (v2.3), METLIN database, and LuMet Plant 3.0 database. For metabolite content analysis, the variable important in the projection (VIP) method was used to measure the expression patterns of metabolites, with a parameter of *p* < 0.05 and VIP > 1 as criteria for screening differentially expressed metabolites (DEMs).

### 2.3. Transcriptomic Analysis of Isodon Suzhouensis

We used the TRIzol method to extract RNA from *Isodon suzhouensis* according to the manufacturer’s instructions. The quality of the RNA was assessed using the 5300 Bioanalyser (Agilent, Palo Alto, CA, USA) and quantified with the NanoDrop ND-2000 (Thermo Scientific, Waltham, MA, USA). The RNA sample was considered suitable for constructing the sequencing library if it met the following criteria: OD_260/280_ = 1.8~2.2, OD_260/230_ ≥ 2.0, RIN ≥ 6.5, and 28S:18S ≥ 1.0. To prepare the cDNA library, we used 1 μg of total RNA and prepared according to the Illumina^®^ Stranded mRNA Prep (San Diego, CA, USA). The cDNA was then subjected to end-repair, phosphorylation, and ‘A’ base addition following Illumina’s library construction protocol. The libraries were size-selected for cDNA target fragments of 300 bp on 2% Low Range Ultra Agarose and amplified using Phusion DNA polymerase (NEB) for 15 PCR cycles. After quantification with Qubit 4.0, the paired-end RNA-seq sequencing library was sequenced on the Illumina NovaSeq 6000 platform to obtain raw paired-end reads. These raw reads were then analyzed using Fastp (https://github.com/OpenGene/fastp (accessed on 4 July 2023)) to obtain clean reads. The clean reads were then annotated in six databases (NR, SWISS-PROT, KEGG, GO, Eggnog, Pfam) to obtain gene annotation information. Gene expression levels were analyzed using the Fragments Per Kilobase of Exon Model Per Million Mapped Fragments (fpkm) method. The differential expression analysis was performed using DESeq2 (http://bioconductor.org/packages/stats/bioc/DESeq2/ (accessed on 4 July 2023)) with |log_2_FC| ≥ 1 and FDR ≤ 0.05 (DESeq2). In this study, the annotated genes were used for *KS* gene mining.

### 2.4. Mining, Cloning, and Bioinformatic Analysis of IsKS Gene from Isodon suzhouensis

Unigenes annotated to the diterpenoid biosynthesis pathway (map00904) were identified. Among them, unigenes annotated as ent-kaurene synthase (EC:4.2.3.19) were selected and compared with *KS* from *Arabidopsis thaliana* (https://www.arabidopsis.org/ (accessed on 20 December 2023)) and maize (https://db.cngb.org/ (accessed on 20 December 2023)). The amino acid sequence of IsKS was compared using DNAMAN (version 8.0). Conserved motifs of IsKS were identified using MEME online software (https://meme-suite.org/meme/ (accessed on 20 December 2023)). The phylogenetic tree of IsKS was constructed using Clustal X (version 1.8) and MEGA (version 6.0). For cloning the *IsKS* gene, we extracted total RNA from *Isodon suzhouensis* using the RNAprep Pure Plant Plus Kit (Tiangen, Beijing, China), synthesized first-strand cDNA using the TIANScript II RT Kit (Tiangen, Beijing, China), and amplified *IsKS* using primers KS-F (ATGTCTCTTATTCTCTCCTCTTTC) and KS-R (TTAGAGGTAAAGATTTGGCAGATGG). The PCR system and reaction conditions followed the Taq DNA Polymerase instructions, with an annealing temperature of 60 °C. The PCR products were then ligated into pMD18-T vectors and transformed into *E. coli* DH5α competent cells and sequenced. For bioinformatic analysis, the amino acid sequence of IsKS was submitted to NCBI (https://www.ncbi.nlm.nih.gov/cdd (accessed on 2 February 2024)) to identify conserved domains, to Swiss-Model (https://www.swissmodel.expasy.org/ (accessed on 2 February 2024)) to predict the tertiary structure, and to SOPMA (https://npsa.lyon.inserm.fr/cgi-bin/npsa_automat.pl?page=/NPSA/npsa_sopma.html (accessed on 2 February 2024)) to predict the secondary structure of protein. Furthermore, the amino acid sequence of IsKS was compared with KS from *Arabidopsis thaliana*, and the substrate binding pocket and Mg^2+^ binding sites in IsKS were also predicted.

### 2.5. Construction of Recombinant Plasmid for IsKS Gene Overexpression and Subcellular Location

To construct the recombinant plasmid for the overexpression of the *IsKS* gene, we utilized primers KS-F1 (AGAGAACACGGGGACTTTGCAACATGTCTCTTATTCTCT CCTCTTTCTCACTTTTTC) and KS-R1 (TTCCTCGCCCTTCACGATACACATGAG GTAAAGATTTGGCAGATGGTACTCA) for cloning the *IsKS* gene into the eukaryotic plasmid pBWA(V)BS-GFP-GUS through homologous recombination method, resulted in the formation of the plasmid pBWA(V)BS-*KS*-GFP-GUS. The recombinant plasmid was then transiently transformed into GV3101-competent cells and injected into tobacco leaves, with the empty plasmid pBWA(V)BS-GFP-GUS serving as a positive control. After two days of growth in the dark, the tobacco leaves were examined using a laser confocal microscope with an excitation wavelength of 488 nm.

### 2.6. Molecular Docking of the IsKS1 and the Substrates ent-CPP

The protein sequence of IsKS1 was submitted to Swiss-Model (https://www.swissmodel.expasy.org/ (accessed on 20 June 2024)) for the prediction of its tertiary structure. The tertiary structure of IsKS1 was then downloaded and converted to the pdb format using Chem3D software (version 20.0.0.41). After the addition of hydrogen, the IsKS1 protein was exported in pdbqt format using AutoDockTools (version 1.5.7). The structure of the substrate, ent-copalyl diphosphate (ent-CPP), was drawn using ChemDraw (version 20.0.0.41), saved as a pdb file, and then converted to pdbqt using AutoDockTools. The docking of ent-CPP and IsKS1 was performed using AutoDockTools, with the docking parameters set as a genetic algorithm (GA) and the number of GA runs set to 100. All potential binding sites of ent-CPP and IsKS1 were viewed using the protein–ligand interaction profiler (https://plip-tool.biotec.tu-dresden.de/ (accessed on 20 June 2024)).

### 2.7. Overexpression of the IsKS1 Gene in Arabidopsis and Analysis of Arabidopsis Morphology

The recombinant plasmid pBWA(V)BS-*KS*-GFP-GUS was introduced into GV3101 and transformed into *Arabidopsis thaliana* using the floral dip method. After two months of growth, the seeds were harvested and screened with glufosinate ammonium. The well-grown Arabidopsis seeds were then identified by PCR using primers designed from the *bar* gene (F: CGGCGA CGAGCCAGGGATA; R: GCACCATCGTCAACCACTACAT). For PCR identification, gDNA was isolated from *Arabidopsis thaliana* using the CTAB method, and the PCR reaction conditions were performed according to the Taq DNA Polymerase instructions with an annealing temperature set at 50 °C. Furthermore, GUS staining was performed on various tissues (flowers, leaves, pods, and roots) of *Arabidopsis thaliana* using a GUS staining kit (Solarbio, Beijing, China). To investigate the potential effects of the *IsKS1* gene on gibberellin biosynthesis and any resulting morphological changes, the flowering time, germination rate, and root length of the T3 generation of Arabidopsis were analyzed.

### 2.8. Metabolomic and Transcriptomic Analysis of IsKS1 Gene Overexpression Arabidopsis thaliana

Wild-type and *IsKS1*-overexpressing *Arabidopsis thaliana*, both grown for 35 days, were subjected to LC-MS/MS analysis. The analytical methods were identical to those used for *Isodon suzhouensis*. Principal component analysis (PCA) of metabolite data was performed, and metabolites with VIP scores greater than 1 and *p*-values less than 0.05 were identified as differentially expressed metabolites (DEMs). Kyoto Encyclopedia of Genes and Genomes (KEGG) enrichment analysis of the DEMs was performed, and DEMs in the diterpenoid biosynthetic pathway (map00904) were identified to assess the impact of the *IsKS1* gene on gibberellin (GA) biosynthesis. Furthermore, we also conducted a transcriptomic analysis of the T3 generation of transgenetic *Arabidopsis thaliana* and analyzed the gene expression level in map00904.

### 2.9. Statistical Analysis

Statistical analyses were performed using Excel (version 14). Analysis of variance was used to compare the statistical deference based on Student’s *t*-test, with the level of significance set at *p* < 0.05, and data were presented as mean ± S.D.

## 3. Results

### 3.1. Differential Metabolites Present in the Leaves and Stems of Isodon suzhouensis

**Figure 1:**
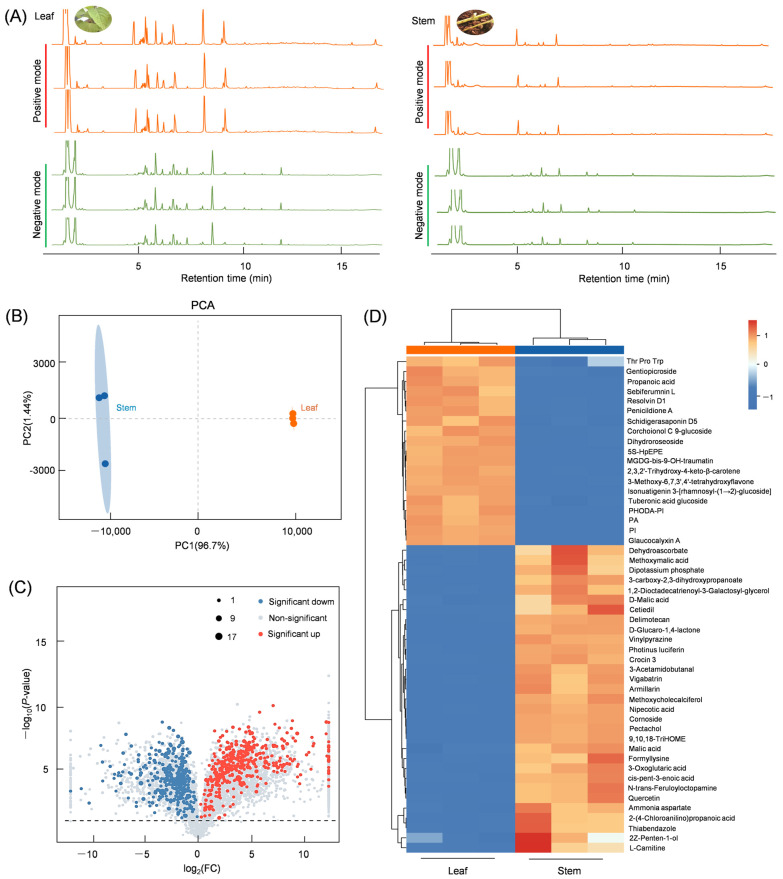
Quality assessment of DEMs in the leaf and stem of *Isodon suzhouensis*. (**A**) Total ion chromatogram (TIC) of leaf and stem samples in both positive and negative EI modes. (**B**) Principal component analysis (PCA) of leaf and stem samples. (**C**) Volcano plot of DEMs in leaf and stem. (**D**) Heat map of the top enriched DEMs.

*Isodon suzhouensis* primarily utilizes its leaves for medicinal purposes, and they are also commonly used as tea for their health benefits. In order to investigate the reasons for this, we conducted a comparison of metabolites between leaf and stem samples of *Isodon suzhouensis* using mass spectrometry analysis. Both positive and negative modes were utilized for UPLC-MS/MS analysis, and compounds were identified by comparing MS data with those of the database. The total ion chromatogram (TIC) revealed a higher abundance of metabolites in the leaves compared to the stems, indicating the significance of leaves as both medicinal and edible materials (Figure 1A). The quality of metabolic data from the leaf and stem tissues was evaluated using Principal Component Analysis (PCA). The tissue samples, whether leaf or stem, were tightly clustered, demonstrating the consistency of metabolites in these samples and highlighting significant differences between the metabolites in the leaves and stems (Figure 1B). Metabolite levels were calculated and compared using VIP, FC, and *p*-values, with compounds having a *p*-value < 0.05 and VIP > 1.0 considered to have significantly different levels. After screening, 767 DEMs were identified between leaves and stems, with 411 compounds showing significantly higher levels in the leaves (Figure 1C). Cluster analysis of the DEMs led to the identification of compounds such as glaucocalyxin A, gentiopicroside, sebiferumnin L, resolvin D1, schidigerasaponin D5, corchoionol C-9 glucoside, isonuatigenin 3-glucoside, and tuberonic acid glucoside, among others. (Figure 1D). The enrichment results of these metabolites provide insight into the use of leaves as medicinal tissues and have prompted further analysis of gene expression in the biosynthetic pathways of these metabolites.

### 3.2. KEGG Enrichment of DEMs

**Figure 2:**
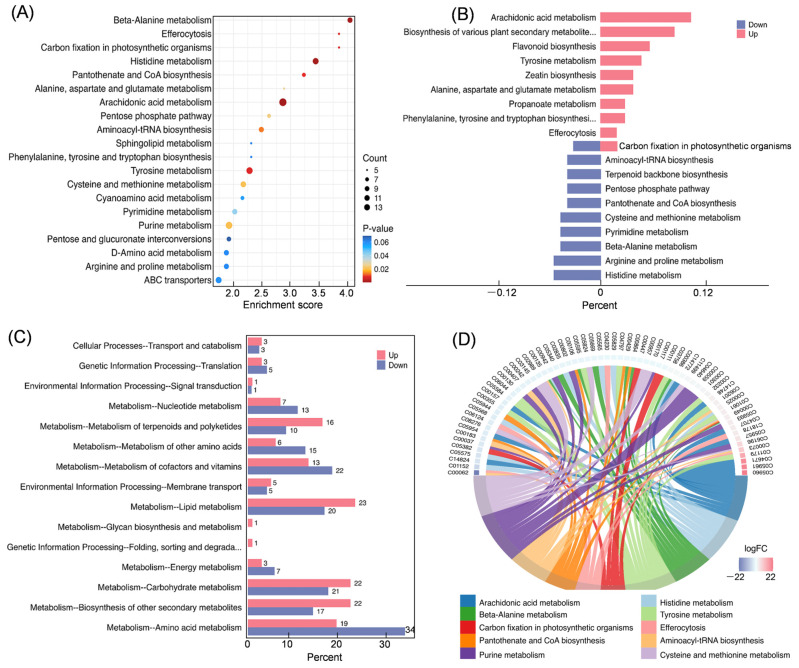
Enrichment analysis of DEMs in leaf and stem of *Isodon suzhouensis*. (**A**) The top enriched KEGG pathway of DEMs. (**B**) The enriched KEGG pathways of up- and down-regulated DEMs. (**C**) The KEGG pathway classification of the annotated DEMs. (**D**) The chord of significantly enriched DEMs.

Analysis of the DEMs enrichment pathways revealed that arachidonic acid metabolism (map00590) was significantly enriched, with 14 components enriched in this pathway. This was followed by purine metabolism (map00230), tyrosine metabolism (map00350), ABC transporters (map02010), and histidine metabolism (map00340), which contained 12, 11, 10, and 10 metabolites, respectively (Figure 2A). The up-regulated pathways in leaves included arachidonic acid metabolism, alanine, aspartate, and glutamate metabolism, flavonoid biosynthesis, zeatin biosynthesis, tyrosine metabolism, phenylalanine, tyrosine and tryptophan biosynthesis, propanoate metabolism, photosynthetic carbon fixation, and efferocytosis. Conversely, the down-regulated pathways included beta-alanine metabolism, histidine metabolism, pantothenate and CoA biosynthesis, carbon fixation in photosynthetic organisms, pentose phosphate pathway, arginine and proline metabolism, terpenoid backbone biosynthesis, pyrimidine metabolism, cysteine and methionine metabolism, and aminoacyl-tRNA biosynthesis. The pathways annotated for different metabolites in leaves and stems showed overlap, which can be attributed to the different functions of these pathways or metabolites in growth processes (Figure 2B). Among these pathways, those with the highest number of annotated DEMs were amino acid metabolism, lipid metabolism, and carbohydrate metabolism (Figure 2C). Analysis of significantly up- and down-regulated metabolites showed that the significantly up-regulated metabolites in leaves were 15-oxoprostaglandin F2α, 6-oxoprostaglandin F1α, 13,14-dihydro-15-keto-prostaglandin E2, and 4-hydroxyphenylpyruvic acid. The significantly down-regulated metabolites in leaves were L-arginine, 3-methyl-L-histidine, and 8(R)-hydroxy-(5Z,9E,11Z,14Z)- eicosatetraenoic acid (Figure 2D).

### 3.3. Transcriptomic Data from Leaf and Stem of Isodon suzhouensis

**Figure 3:**
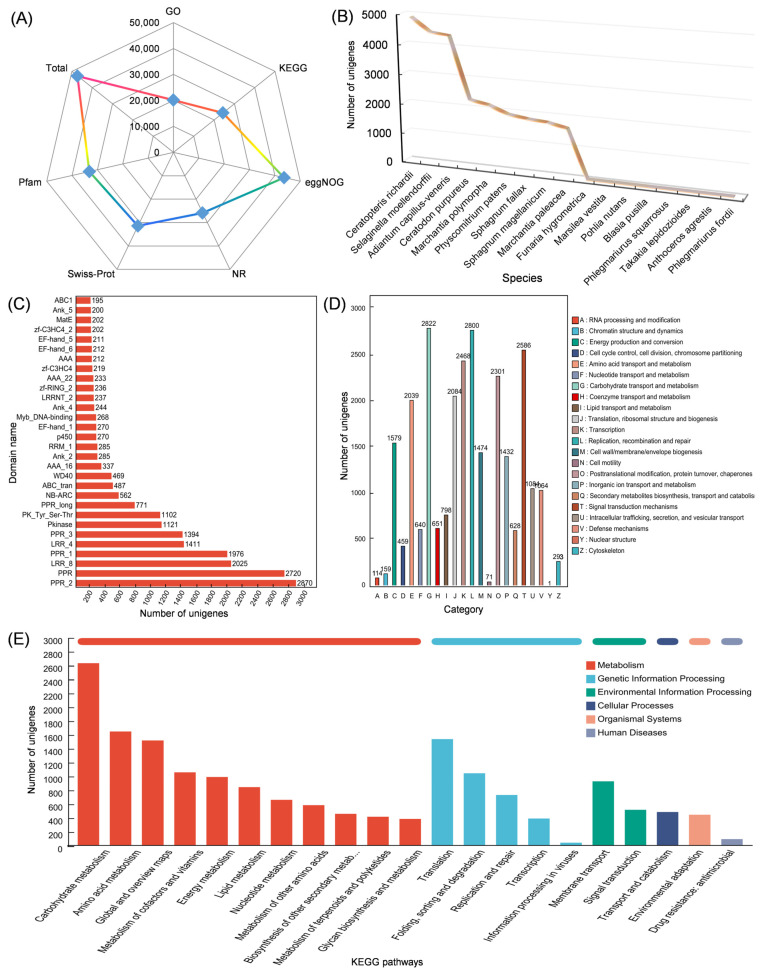
Annotation of unigenes of *Isodon suzhouensis*. (**A**) Functional annotation of the unigenes in the EggNOG, NR, Pfam, Swissprot, KEGG, and GO databases. (**B**) Species distribution of the unigenes. (**C**) The top 30 domains contained within the unigenes. (**D**) EggNOG annotation of the unigenes. (**E**) Kyoto Encyclopedia of Genes and Genomes (KEGG) categories of the unigenes.

After concatenating the sequence and removing duplicates, a total of 77,091 unigenes were obtained. Of these, 47,149 were annotated in the EggNOG, NR, Pfam, Swissprot, KEGG, and GO databases (Figure 3A). A high percentage (97.78%) of these unigenes had a hit with nine different species, including *Ceratopteris richardii*, *Selaginella moellendorffii*, *Adiantum capillus-veneris*, *Ceratodon purpureus*, *Marchantia polymorpha*, *Physcomitrium patens*, *Sphagnum fallax*, *Sphagnum magellanicum* and *Marchantia paleacea* (Figure 3B). Further analysis of the domains present in these unigenes revealed that the PPR_2 superfamily domain (pfam13041) and the pentatricopeptide repeat (PPR) domain were the most common, followed by the leucine-rich repeat (LRR) domain and the protein kinase domain (pkinase) (Figure 3C). EggNOG annotation showed that 2822 unigenes were functionally annotated in carbohydrate transport and metabolism, with replication, recombination, and repair being the second most common function (2800 unigenes). Other common functions included signal transduction mechanisms (2586 unigenes), transcription (2468 unigenes) and post-translational modification, protein turnover, and chaperones (2301 unigenes) (Figure 3D). KEGG annotation revealed that the majority of the unigenes were involved in carbohydrate metabolism (2637 unigenes), followed by amino acid metabolism (1651 unigenes), translation (1542 unigenes), global and overview maps (1523 unigenes), metabolism of cofactors and vitamins (1062 unigenes) and folding, sorting and degradation (1048 unigenes) (Figure 3E).

### 3.4. Differential Expression Gene Analysis

**Figure 4:**
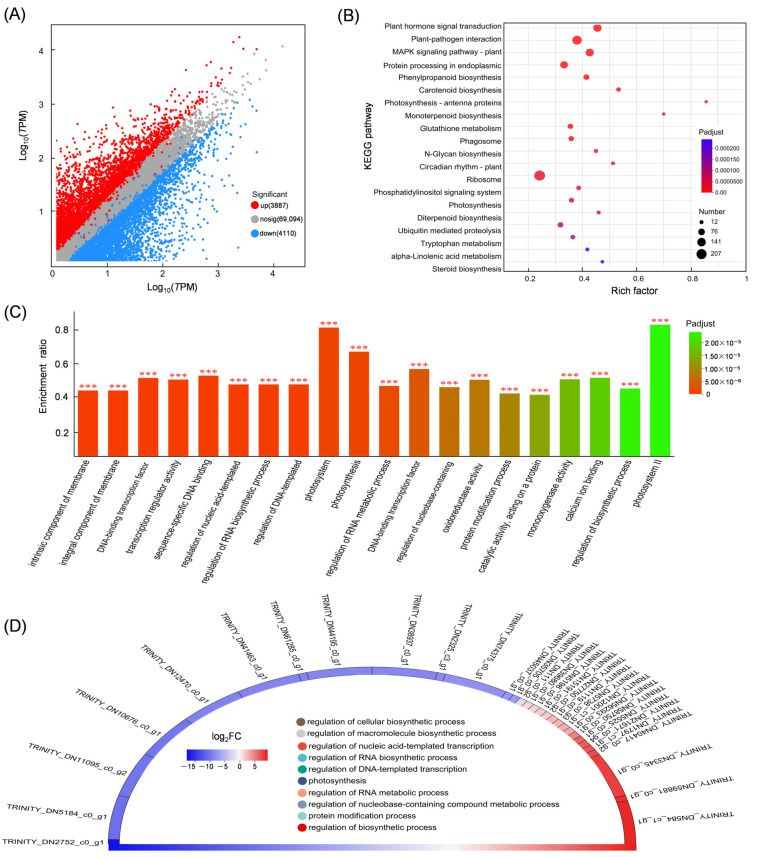
Analysis of DEGs in the leaf and stem of *Isodon suzhouensis*. (**A**) Scatter plot of DEGs. (**B**) KEGG enrichment of DEGs. (**C**) GO enrichment of DEGs. (**D**) Chord diagram of enriched DEGs. *** *p*-adjust < 0.001.

A total of 7997 differentially expressed genes (DEGs) were identified in the leaves and stems of *Isodon suzhouensis*. Of these, 3887 unigenes were exclusively up-regulated in leaves compared to stems, while 4110 unigenes were exclusively down-regulated (Figure 4A). Subsequent KEGG analysis of the DEGs revealed that 207 unigenes were associated with the ribosome pathway (map03010), 157 unigenes with the plant–pathogen interaction pathway (map04626), 118 unigenes with plant hormone signal transduction (map04075), and 118 and 117 unigenes with cofactor biosynthesis (map01240) and the MAPK signalling pathway (map04016), respectively (Figure 4B). GO enrichment analysis revealed that the DEGs were enriched in intrinsic components of the membrane (2217 unigenes), DNA-binding transcription factor activity (366 unigenes), transcriptional regulator activity (397 unigenes), catalytic activity (1136 unigenes) and regulation of biosynthetic processes (363 unigenes) (Figure 4C). Subsequent analysis of the top significant DEGs revealed that TRINITY_DN584_c1_g1 (BEL1-like homeodomain protein), annotated in the regulation of nucleic acid-templated transcription, was significantly up-regulated with a log_2_FC value of 9.4, followed by TRINITY_DN59881_c0_g1 (wall-associated receptor kinase) annotated in protein modification processes with a log_2_FC value of 9.1, and TRINITY_DN3345_c0_g1 (agamous-like MADS box protein) annotated in the regulation of nucleic acid-templated transcription, and TRINITY_DN3345_c0_g1 annotated in regulation of macromolecule biosynthetic processes. The top down-regulated unigenes were TRINITY_DN2752_c0_g1 (agglutinin-2 protein), TRINITY_DN5184_c0_g1 (scarecrow-like protein), and TRINITY_DN11095_c0_g2 (agamous-like MADS box protein AGL11) (Figure 4D).

### 3.5. Mining DEGs Involved in the Diterpenoid Biosynthetic Pathway

Wangzaozins (wangzaozin A, glaucocalyxin A, and glaucocalyxin B) are tetracyclic diterpenoids whose structure was elucidated in *Isodon suzhouensis*. The biosynthesis of wangzaozins follows the diterpenoid biosynthetic pathway (map00904). After annotating the genes, a total of 41 DEGs were identified in map00904. These genes included five unigenes identified as copalyl diphosphate synthase (*CPS*), six unigenes identified as ent-kaurene synthase (*KS*), and P450 enzyme genes such as gibberellin 3-β-dioxygenase (*GA2ox*), ent-kaurene oxidase (*GA3*), gibberellin 20 oxidase (*GA20ox*), and ferruginol synthase (Table 1). In this study, the expression levels of the genes TRINITY_DN2848_c0_g1 and TRINITY_DN9009_c0_g1, annotated as kaurene synthase, were examined in leaf and stem samples. The results showed that these two genes had higher expression levels in leaf samples than in stem samples. This finding suggests that they may play a role in terpenoid skeleton synthesis and wangzaozin accumulation.

### 3.6. Identification of Two IsKS Genes from Isodon suzhouensis

**Figure 5:**
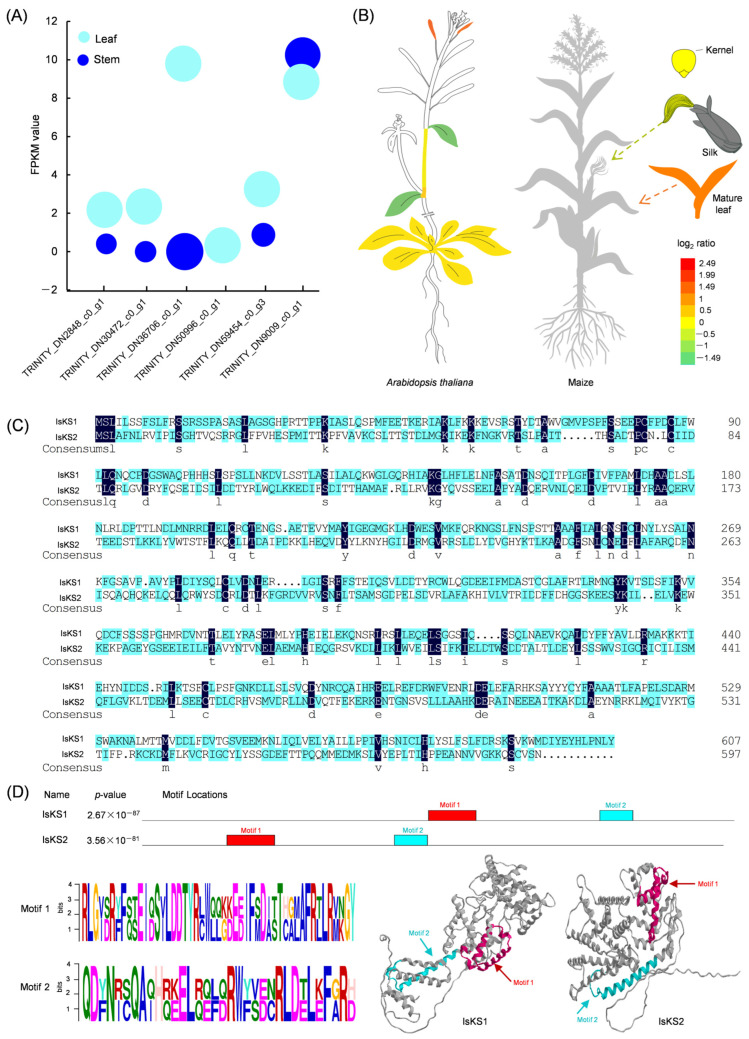
Identification of two *IsKS* from *Isodon suzhouensis*. (**A**) The relative expression level of *KS* genes in the leaf and stem of *Isodon suzhouensis*. (**B**) The expression level of the *KS* gene in *Arabidopsis thaliana* and maize. (**C**) Alignment of the amino acid sequences of IsKS1 and IsKS2. (**D**) Identification of two conserved motifs of IsKS1 and IsKS2.

A total of six unigenes were identified as ent-atiserene synthase or kaurene synthase (KS):TRINITY_DN30472_c0_g1, TRINITY_DN59454_c0_g3, TRINITY_DN36706_c0_g1, TRINITY_DN50996_c0_g1, TRINITY_DN2848_c0_g1, and TRINITY_DN9009_c0_g1. These unigenes showed up-regulated expression levels in leaves (Figure 5A). The expression pattern of the *IsKS* genes was similar to that of *A. thaliana* (*KS* gene ID AT1G79460.1) and maize (*KS* gene ID Zm00001d002350) (Figure 5B). Among these six unigenes, only TRINITY_DN2848_c0_g1 and TRINITY_DN9009_c0_g1 had full-length ORFs with gene lengths longer than 1000 bp. These two genes were named *IsKS1* and *IsKS2*, respectively. Amino acid sequence analysis showed that IsKS1 and IsKS2 shared 11.74% identity (Figure 5C). Additionally, both IsKS1 and IsKS2 contained a conserved domain with two distinct motifs. Motif 1 had the amino acid sequence ‘RLGIDRFFQSEIDSILDDTYRCWQQGDEEIFMDISTCGLAFRLLRMKGY’, while motif 2 had the amino acid sequence ‘QDFNR CQAQHREELRZFDRWFVECRLDELEFGRD’ (Figure 5D).

### 3.7. Cloning, Bioinformatic Analysis, and Subcellular Localization of IsKS1

**Figure 6:**
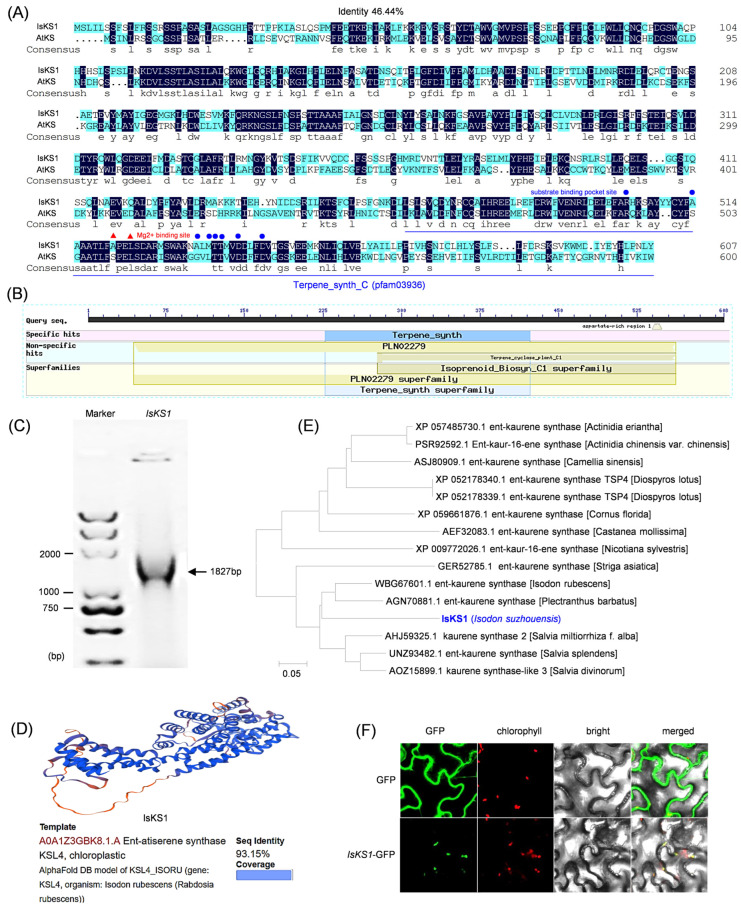
Cloning and bioinformatic analysis of IsKS1. (**A**) Comparison of the amino acid sequence of IsKS1 with that of *Arabidopsis thaliana*. (**B**) Analysis of the conserved domain of IsKS1. (**C**) Agarose electrophoresis analysis of *IsKS1*. (**D**) Tertiary structure of IsKS1. (**E**) Phylogenetic analysis of IsKS1 with KS from other species. (**F**) Subcellular localization of IsKS1.

*IaKS1*, which had a higher expression level in the leaf than in the stem, was cloned from *Isodon suzhouensis*. Further bioinformatic analysis was conducted, revealing that *IsKS1* has a gene length of 1827 bp and encodes an amino acid sequence of 608 aa. A comparison of the amino acid sequence and domain of AtKS1 (gene ID AT1G79460.1) with IsKS1 showed that IsKS1 contains the domains PLN02279 (accession PLN02279), terpene_cyclase_plant_C1 (accession cd00684), and terpene_synth_C (pfam03936). The presence of substrate binding pocket sites and Mg^2+^ binding sites suggests potential enzymatic activity of IsKS1 (Figure 6A–C). The physicochemical properties of IsKS1 were analyzed using online informatics analysis tools, revealing a formula of C_3088_H_4771_N_813_O_921_S_31_ and a molecular weight of 69015.61 Da. The theoretical isoelectric point (PI) of IsKS1 was predicted to be 5.52. The grand average of hydropathicity (GRAVY) of IsKS1 was −0.191, indicating that IsKS1 is a hydrophilic protein. The instability index of IsKS1 was calculated to be 55.61, suggesting that the protein is unstable. Secondary structure prediction showed that IsKS1 is composed of 65.13% alpha helix, 2.63% extended strand, 2.30% beta-turn, and 29.93% random coil (Supplementary Materials Figures S1 and S2). The tertiary structure revealed that IsKS1 shares 93.15% similarity with the ent-atiserene synthase KSL4 (accession number A0A1Z3GBK8.1.A) from *Isodon rubescens* (*Rabdosia rubescens*), indicating potential similar biological activity (Figure 6D). Phylogenetic analysis showed that IsKS1 has a closer evolutionary relationship with KS from *Isodon rubescens* and *Plectranthus barbatus* (Figure 6E). To determine the subcellular localization of IsKS1 and for further genetic transformation in *Arabidopsis thaliana*, we constructed the fusion plasmid pBWA(V)BS-KS-GFP-GUS and used it for the transient transformation of tobacco leaves (Supplementary Materials Figure S3). The fluorescence signal was observed using a laser confocal microscope, with green fluorescence mainly observed in the chloroplasts of tobacco leaves transformed with *KS*-GFP-GUS, consistent with the natural fluorescence of chloroplasts. The subcellular localization results confirmed that IsKS1 is located in the chloroplasts, as predicted by PlantPloc (Figure 6F).

### 3.8. Docking Analysis of IsKS1 with ent-CPP

**Figure 7:**
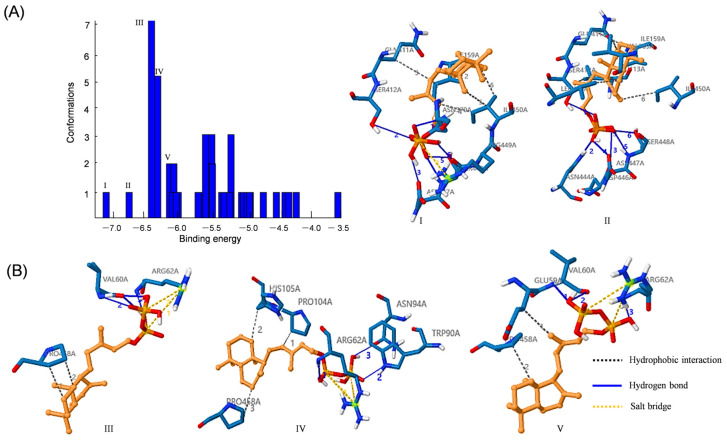
Docking analysis of IsKS1 with ent-CPP. (**A**) The predicted binding energy of ent-CPP to the IsKS1 protein. (**B**) The top five lowest-energy binding modes of ent-CPP to IsKS1. I–V, represent the five binding modes between substrates ent-CPP and IsKS1.

To assess the enzymatic activity of IsKS1, we utilized AutoDock software to predict the potential binding sites of IsKS1 to the substrate ent-CPP (ent-CPP). This resulted in 20 favourable substrate-binding conformations (Figure 7A). From these, we analyzed the top five potential binding modes between the protein IsKS1 and the substrate ent-CPP with the lowest energy. For example, in binding mode I, hydrophobic interactions were observed between amino acid residues of ILE159, GLN411, ILE450, and C1-C4 of ent-CPP. Additionally, hydrogen bonds were also observed between the phosphate group of ent-CPP and ASN370, SER412, ASP447, and SER448. Furthermore, salt bridges were observed between ARG449 and the phosphorus atom of ent-CPP (Figure 7B). These findings suggest that IsKS1 binds to the substrate ent-CPP, indicating a potential catalytic activity of IsKS1 in *Isodon suzhouensis*.

### 3.9. Overexpression of IsKS1 Arabidopsis thaliana and Phenotypic Analysis

**Figure 8:**
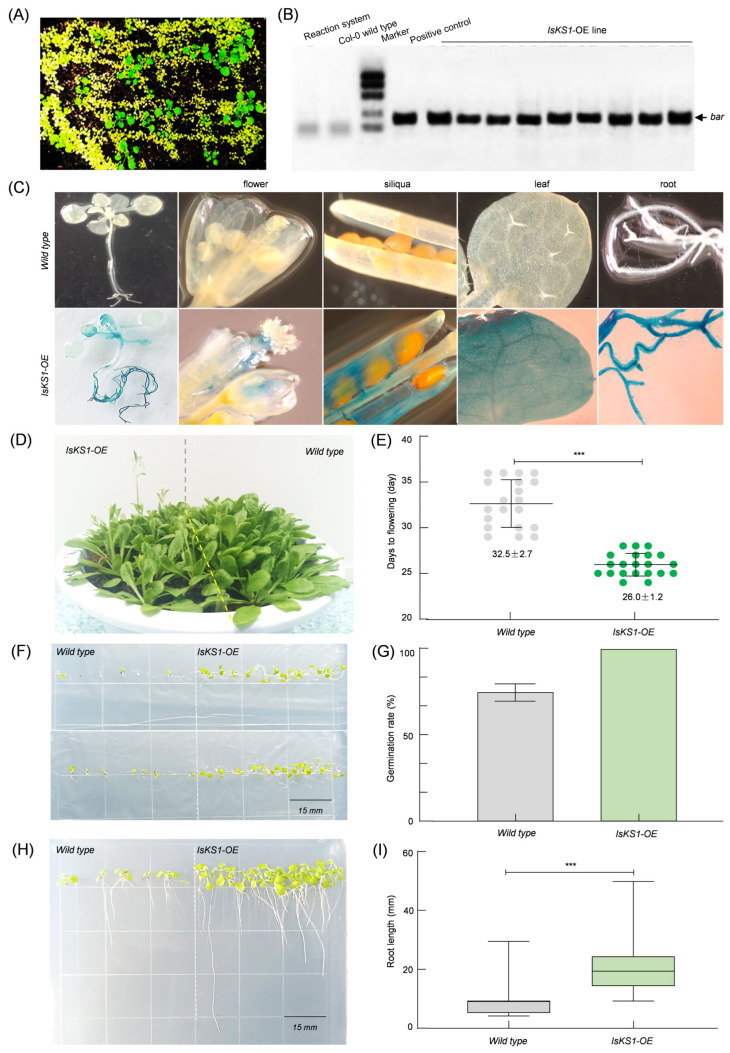
Identification and phenotypic analysis of *IsKS1*-overexpressing *Arabidopsis thaliana*. (**A**) Screening of *IsKS1*-overexpressing *Arabidopsis thaliana* with glufosinate ammonium. (**B**) PCR screening of *IsKS1* overexpression lines by *bar* gene detection. (**C**) GUS staining of *IsKS1* overexpression lines. (**D**) Phenotypic analysis of wild-type and *IsKS1*-overexpressing *Arabidopsis thaliana* 35 days after sowing. (**E**) Days to the flowering of wild-type and *IsKS1*-overexpressing *Arabidopsis thaliana*. (**F**) Phenotypic analysis of *Arabidopsis thaliana* after sowing on 1/2 MS medium for 5 days. (**G**) The germination rate of *Arabidopsis thaliana* after 5 days of sowing. (**H**) The phenotype of *Arabidopsis thaliana* after sowing on 1/2 MS medium for 10 days. (**I**) Root length of *Arabidopsis thaliana* after 10 days of sowing.*** *p*≤ 0.001, *n* = 20.

The *IsKS1* gene was overexpressed in *Arabidopsis thaliana* using the floral dip method. Successful transformants were identified by spraying them with herbicide (glufosinate ammonium). The transgenic *IsKS1* lines showed robust growth due to the presence of the *bar* gene, which confers resistance to the herbicide (Figure 8A). This was subsequently confirmed using a PCR method based on the *bar* gene. All successful transformants showed a band on agarose gel electrophoresis analysis, while the wild-type lines did not (Figure 8B). Further analysis of *IsKS1* gene expression in different tissues using GUS staining revealed the presence of blue staining in all *Arabidopsis* tissues, indicating that *IsKS1* has no tissue-specific expression (Figure 8C). Previous research has shown that the *KS* gene is involved in the biosynthesis of gibberellin, a plant hormone that promotes plant growth. To verify whether the *IsKS1* gene has the same effect, we observed the growth status of transgenic *Arabidopsis thaliana* and found that the wild-type took longer to flower (32 days), whereas the *IsKS1*-overexpressing lines reached flowering in only 26 days. Additionally, the transgenic lines had a 100% germination rate and longer root length (21.2 mm) compared to the wild-type (9.9 mm), suggesting that the *IsKS1* gene promotes growth and early flowering in *Arabidopsis thaliana*. These findings can be attributed to the understood role of the *IsKS1* gene in gibberellin synthesis. Further metabolite identification is required to fully understand this relationship (Figure 8D–I).

### 3.10. Detection of GAs and DEGs in the Diterpenoid Biosynthesis Pathway in IsKS1- Overexpressing Arabidopsis thaliana

**Figure 9:**
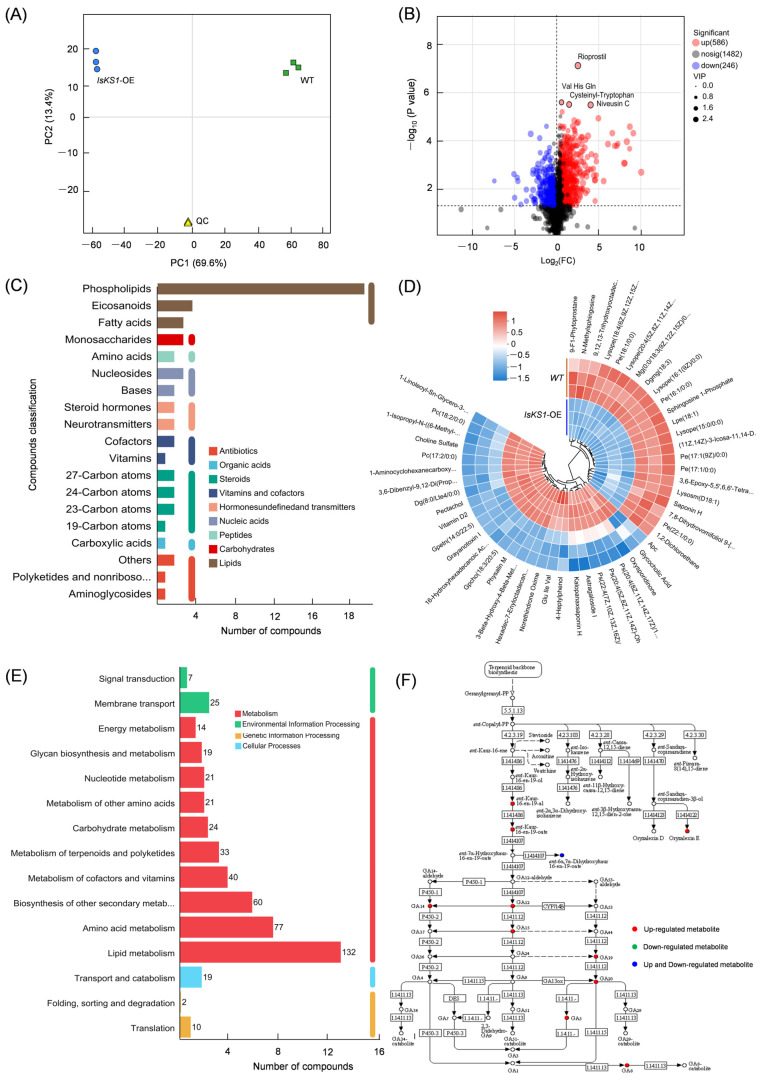
Metabolomic analysis of *IsKS1*-overexpressing *Arabidopsis thaliana*. (**A**) Principal component analysis (PCA) of wild-type and *IsKS1*-overexpressing *Arabidopsis thaliana*. (**B**) Volcano plot of DEMs in the *IsKS1*-overexpressing lines versus wild-type lines. (**C**) Types of DEMs. (**D**) Cluster tree diagram of the top up- and down-regulated DEMs. (**E**) The annotated KEGG pathway of DEMs. (**F**) Expression levels of DEMs annotated in the diterpenoid biosynthesis pathway.

Metabolites were detected in both wild-type and *IsKS1* gene overexpression lines. PCA analysis of metabolites in either wild-type or *IsKS1*-OE samples showed a strong correlation (Figure 9A). A total of 2321 compounds and 832 DEMs were detected, with 586 metabolites up-regulated in the *IsKS1* overexpression lines (Figure 9B). These DEMs included phospholipids, monosaccharides, steroid hormones, 27 carbon atoms, polyketides, non-ribosomal peptides, and aminoglycosides (Figure 9C). The most significantly up-regulated compounds in the *IsKS1* gene overexpression lines were 1-leinoleoyl-Sn-glycoro-3-phosphatoline, pectachol, grayanotoxin I, oxysporidinone, and others (Figure 9D). KEGG pathway analysis of these annotated DEMs showed that lipid metabolism was the most abundantly annotated pathway, followed by amino acid metabolism, cofactor, and vitamin metabolism, terpenoid, and polyketide metabolism (Figure 9E). Among these DEMs, 13 compounds annotated in the diterpenoid pathway were identified, including ent-Kaur-16-En-19-al, ent-Kaur-16-En-19-oate, GA5, GA8, GA12, GA14, and GA15, all of which were up-regulated in *IsKS1* overexpression lines (Figure 9F). Further analysis of the genes involved in the diterpenoid biosynthesis pathway in the transgenic *Arabidopsis* revealed that two genes were up-regulated: Gibberellin 3-beta-dioxygenase 1 (AT1G15550) and Ent-kaur-16-ene synthase (KS, AT1G79460) (Supplementary Materials Table S1). This suggests that the heterologous overexpression of *IsKS* from *Isodon suzhouensis* may induce the up-regulation of *AtKS*, leading to the accumulation of GAs. The metabolomic analysis and gene expression analysis in the diterpenoid biosynthesis pathway of the *IsKS1* gene overexpression in *Arabidopsis thaliana* demonstrated the likely involvement of *IsKS1* in gibberellin skeleton synthesis.

## 4. Discussion

Terpenoid metabolites are bioactive substances produced by plants. An increasing number of novel terpenoid constituents with unique biological activities have been discovered in medicinal plants. The Lamiaceae family of medicinal plants has a long history of use in traditional Chinese medicine due to its wide range of applications, either as food or medicine and its abundant resources. Modern research has shown that *Lamiaceae* plants are rich in diterpenoids, most of which exhibit significant bioactivity. In the last decade, researchers worldwide have reported 246 new diterpenoid compounds isolated from *Lamiaceae* medicinal plants. Examples include scubatin (A–F) isolated from *Scutellaria barbata* [10], splendidin A–C from *Salvia hispanica* and *Salvia splendens* [11,12], acetoxyhardwickiic acid from *Salvia adenophora Fernald* [13], rubesanolide C–E from *Isodon rubescens* [14], laxiflorin from *Isodon eriocalyx* [15] and phyllostachysin from *Isodon phyllostachys* [16]. These terpenoids have been shown to possess anti-tumor, anti-inflammatory, and hypoglycemic properties, suggesting potential for further development and providing new opportunities for the identification of new pharmaceutical agents.

*Isodon suzhouensis*, a plant from the Lamiaceae family, is highly valued for its medicinal properties. It has a well-developed root system, and its leaves are suitable for making tea. In vitro and in vivo studies have demonstrated that extracts of *Isodon suzhouensis* possess a wide range of pharmacological activities, including anti-tumor, antioxidant, antibacterial, anti-inflammatory, antithrombotic, and anticancer effects. The presence of terpenoid compounds, specifically the diterpenoids wangzaozin A, glaucocalyxin A, glaucocalyxin B, and oleanolic acid, has been identified as the primary factor contributing to these beneficial properties. The diterpene backbone of the wangzaozins is synthesized via the diterpene biosynthetic pathway, which shares the same biosynthetic steps as the plant hormone gibberellin (GA). The *CPS* and *KS* genes have been shown to be involved in GA biosynthesis.

The precursor for gibberellin (GA) biosynthesis is geranylgeranyl diphosphate (GGDP), which is catalyzed by CPS and KS to form ent-kaurene. The ent-kaurene is then converted to GA12 by the action of cytochrome P450 enzymes. GA12 is then further converted by GA3 oxidase (GA3OX) and GA20 oxidase (GA20OX) to other derivatives including GA15, GA1, GA3, and GA4. CPS and KS are considered to be the rate-limiting enzymes in GA biosynthesis. The expression of *CPS* is primarily regulated by developmental cues, with higher levels observed in rapidly growing tissues [17,18]. Conversely, the expression of *KS* is constitutive, with elevated levels observed in developing tissues such as leaf tips and cotyledons [19]. In this study, we analyzed the expression of six *KS* genes identified in *Isodon suzhouensis* and found that they showed higher expression levels in leaves compared to stems, which is consistent with the expression patterns of *KS* in *Arabidopsis thaliana* and maize.

Flowering is a crucial stage in the plant development cycle, which is regulated by a complex network that integrates environmental signals, such as photoperiod, with endogenous pathways. One of the central pathways involved in this process is gibberellin (GA) signalling, which involves the GA receptor GID1 (GIBBERELLIN INSENSITIVE DWARF1) and the DELLA repressors [20]. Recent studies have revealed that DELLA proteins can influence GA-responsive gene expression by interacting with transcription factors as either co-repressors or co-activators [21]. Under long day (LD) conditions, GA promotes flowering by stimulating the transcription of FLOWERING LOCUS T (FT). Additionally, the application of exogenous GA has been shown to significantly induce male flower development [22,23]. The present study has demonstrated that overexpression of *IsKS1* genes in *A. thaliana* results in flowering five days earlier than in wild-type plants, as well as increased germination rate and root length. Furthermore, in *IsKS1*-overexpressing Arabidopsis thaliana, the levels of GAs and genes such as *GA3OX1* and *GA2(KS)* were up-regulated, suggesting that *IsKS1* genes from *Isodon suzhouensis* likely play a role in GA biosynthesis, leading to accelerated flowering and growth through GA accumulation.

## 5. Conclusions

The tetracyclic diterpenoid wangzaozin is the primary active compound found in *Isodon suzhouensis*. The enzymes CPS and KS are known to be the limiting factors in the biosynthesis of diterpenoids. In order to identify the *IsKS* genes, we conducted transcriptome and metabolome sequencing of *Isodon suzhouensis*. One of the *IsKS* genes, which showed high expression in leaves, was successfully cloned and genetically transformed into *Arabidopsis thaliana*. Our transgenic experiments revealed that the *IsKS1* gene promotes germination, root growth, and early flowering in *Arabidopsis*. Additionally, it was found to up-regulate gibberellin levels and the expression of *GA2* and *GA3OX1*, indicating that the *IsKS1* gene may play a role in regulating plant growth by influencing gibberellin synthesis.

## Figures and Tables

**Table 1:** Differentially expressed unigenes annotated in the diterpenoid biosynthesis pathway.

Unigene ID	Length	KO_Name	NR_Hit Gene	Swiss-Prot_Description
TRINITY_DN48295_c0_g1	249	CYP76AH1	KAH9573688.1	Ferruginol synthase
TRINITY_DN47553_c0_g4	327	TPS04	KAI5069968.1	Santalene and bergamotene synthase
TRINITY_DN28685_c0_g1	2556	CPS	UPQ49771.1	Copalyl diphosphate synthase
TRINITY_DN29900_c0_g1	1185	CYP82G1	KAH9575006.1	Dimethylnonatriene synthase
TRINITY_DN55358_c0_g2	434	GA20ox	KAG6551271.1	Gibberellin 20 oxidase 1-D
TRINITY_DN8466_c0_g2	1018	GA2ox	KAH8968045.1	Gibberellin 2-beta-dioxygenase
TRINITY_DN9483_c0_g2	314	CPS	UPQ49771.1	Ent-copalyl diphosphate synthase
TRINITY_DN40555_c0_g1	1351	GA20ox	KAI5084607.1	Gibberellin 20 oxidase
TRINITY_DN76952_c0_g1	1698	KAO	BAQ20602.1	Ent-kaurenoic acid oxidase
TRINITY_DN30472_c0_g1	675	KSL2	KAH7437475.1	Ent-atiserene synthase KSL1
TRINITY_DN56804_c0_g1	239	CYP76AH1	KAH9555561.1	Cytochrome P450 76T24
TRINITY_DN50495_c0_g1	446	GA3ox	KAH8945774.1	Gibberellin 3-beta-dioxygenase
TRINITY_DN82414_c0_g1	210	GA20ox	KAH7297002.1	Gibberellin 20 oxidase
TRINITY_DN59454_c0_g3	1200	KSL2	XP_024380401.1	Kaurene synthase like
TRINITY_DN3391_c0_g1	1292	GA2ox	KAG0616228.1	Gibberellin 2-beta-dioxygenase
TRINITY_DN39640_c0_g1	2595	CPS	UPQ49771.1	Ent-copalyl diphosphate synthase
TRINITY_DN71939_c0_g1	1738	CYP76AH1	KAI5058385.1	Ferruginol synthase
TRINITY_DN36706_c0_g1	254	KSL2	APP91796.1	Ent-atiserene synthase KSL1
TRINITY_DN50996_c0_g1	293	KSL2	BAQ20600.1	Ent-atiserene synthase KSL4
TRINITY_DN23337_c0_g1	1308	GA2ox	BAG68569.1	Gibberellin 2-beta-dioxygenase
TRINITY_DN34933_c0_g1	2213	CYP76AH1	KAI5083804.1	Ferruginol synthase
TRINITY_DN2848_c0_g1	3116	KSL2	BAQ20600.1	Ent-atiserene synthase KSL1
TRINITY_DN24082_c0_g1	1423	GA20ox	KAI5084607.1	Gibberellin 20 oxidase
TRINITY_DN19844_c0_g1	2557	GA3	BAQ20601.1	Ent-kaurene oxidase
TRINITY_DN8466_c0_g1	1447	GA2ox	XP_024399557.1	Gibberellin 2-beta-dioxygenase
TRINITY_DN9884_c0_g4	270	CYP76AH1	KAG0553548.1	Ferruginol synthase
TRINITY_DN54990_c0_g3	442	CYP76AH1	KAH8938074.1	Cytochrome P450 76T24
TRINITY_DN9009_c0_g1	2208	KSL2	BAQ20600.1	Miltiradiene synthase KSL2
TRINITY_DN55358_c0_g1	938	GA20ox	KAH7297002.1	Gibberellin 20 oxidase
TRINITY_DN9884_c0_g1	1582	CYP76AH1	KAH8935636.1	Ferruginol synthase
TRINITY_DN2234_c0_g1	1067	CPS	UPQ49771.1	Ent-copalyl diphosphate synthase
TRINITY_DN9884_c1_g2	1645	CYP76AH1	KAI5058385.1	Ferruginol synthase
TRINITY_DN9884_c0_g3	1109	CYP76AH1	KAG0562564.1	Ferruginol synthase
TRINITY_DN58290_c0_g1	1127	GA2ox	KAG0576195.1	Gibberellin 2-beta-dioxygenase
TRINITY_DN56614_c0_g1	336	GA2ox	KAH7297735.1	Gibberellin 2-beta-dioxygenase
TRINITY_DN50495_c0_g2	666	GA3ox	ABX10776.1	Gibberellin 3-beta-dioxygenase
TRINITY_DN2629_c3_g1	1183	GA20ox	KAH7297002.1	Gibberellin 20 oxidase
TRINITY_DN5351_c0_g1	1645	CYP76AH1	KAH8956371.1	Carnosic acid synthase
TRINITY_DN9483_c0_g1	2205	CPS	UPQ49773.1	Ent-copalyl diphosphate synthase
TRINITY_DN416_c0_g1	1925	GA3	KAH7284372.1	Ent-kaurene oxidase
TRINITY_DN36338_c0_g1	1234	GA3ox	ABX10776.1	Gibberellin 3-beta-dioxygenase

## Data Availability

The raw RNA-seq data supporting the conclusions of this article are deposited at NCBI under SRA accession number PRJNA1186997. The raw metabolite data in this article are available for analysis at Majorbio (https://analysis.majorbio.com/metab/species_general/task_id/25dv_3345a1d66v6rdosu090kd3 (accessed on 2 August 2024)).

## Supplementary Materials

The following supporting information can be downloaded at: https://www.mdpi.com/article/10.3390/cimb47060413/s1.
