# Supplementary material for: Heterologous Overexpression and Functional Analysis of the Isodon suzhouensis IsKS1 Gene in Arabidopsis thaliana
Source: Curr Issues Mol Biol. 2025 Jun 3;47(6):413. doi: 10.3390/cimb47060413 (PMC12192304; doi:10.3390/cimb47060413)

Supplementary Figures

Heterologous overexpression and functional analysis of the *Isodon suzhouensis IsKS1* gene in *Arabidopsis thaliana*

Figure S1. Predict hydrophobicity and hydrophilicity of IsKS1

Figure S2. Comparison of the tertiary structures of IsKS1 with KS4 (A0A1Z3GBK 8.1.A) from *Isodon rubescens*.

Figure S3. Constructed eukaryotic overexpression recombinant plasmid pBWA(V)BS-KS-GFP-gus and validated by double enzymatic digestion.

Figure S1.

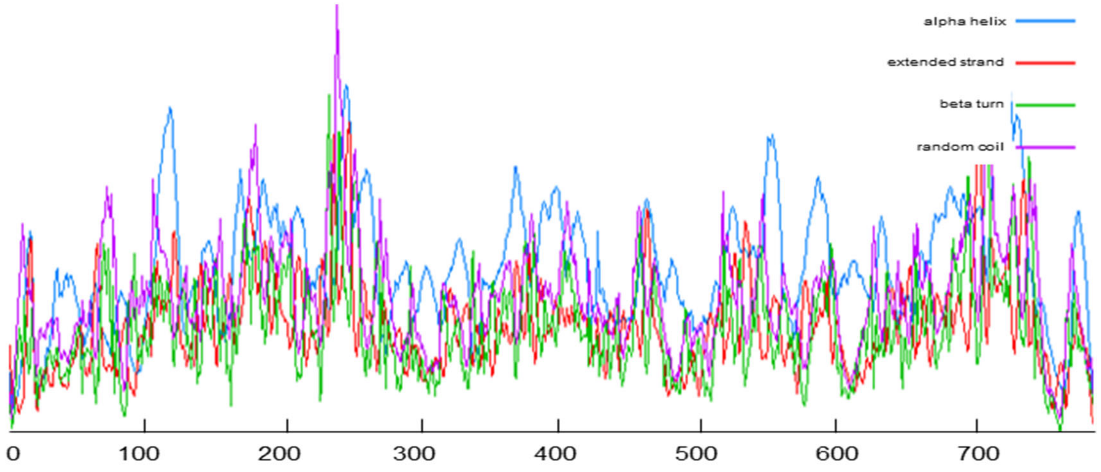

Figure S2.

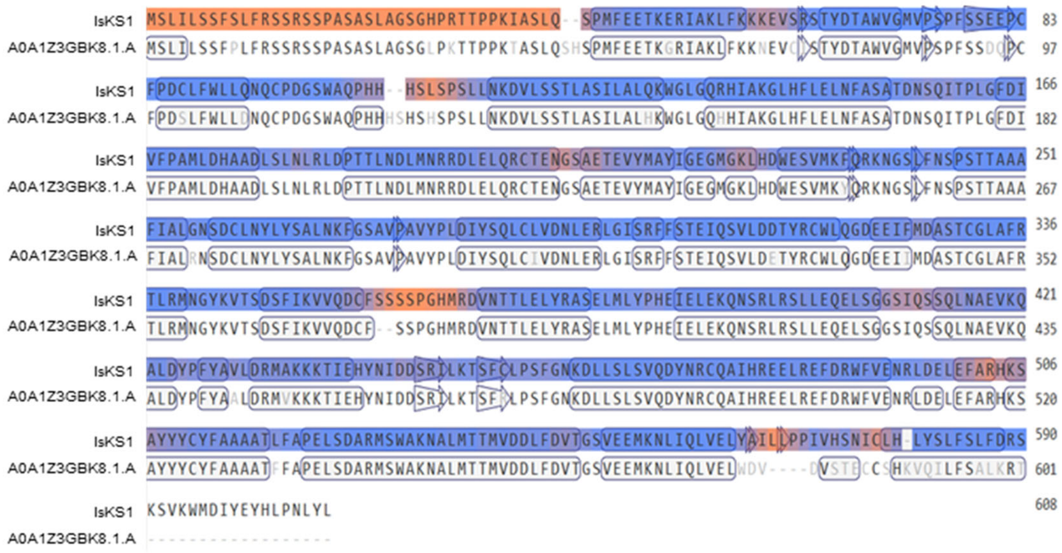

Figure S3.

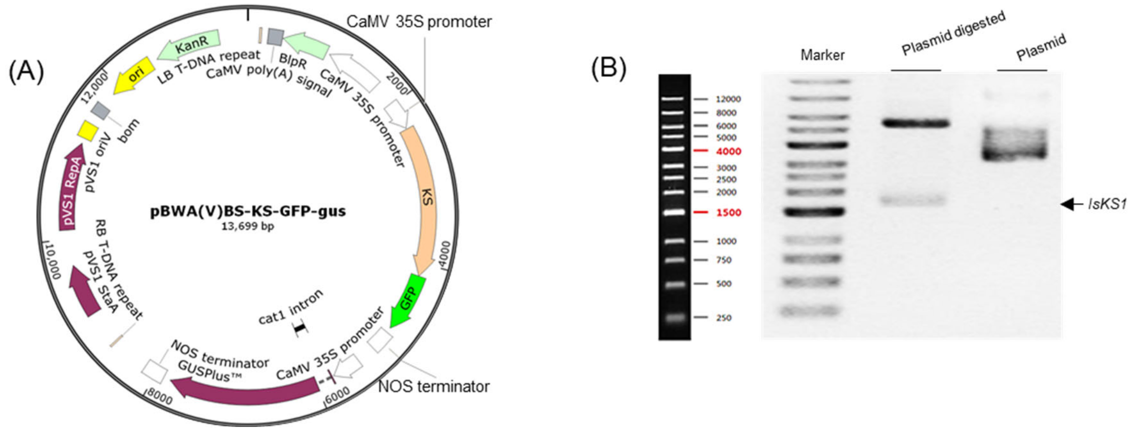

Supplement: Supplementary file 1 [file cimb-47-00413-s001.zip › Supplementary Figures.pdf]
